# Challenges and opportunities associated with the introduction of next-generation long-lasting insecticidal nets for malaria control: a case study from Burkina Faso

**DOI:** 10.1186/s13012-016-0469-4

**Published:** 2016-07-22

**Authors:** Kemi Tesfazghi, Adama Traore, Hilary Ranson, Sagnon N’Fale, Jenny Hill, Eve Worrall

**Affiliations:** 1 Department of Vector Biology, Liverpool School of Tropical Medicine, Pembroke Place, Liverpool, L3 5QA UK; 2 Centre National de Recherche et de Formation sur le Paludisme, BP 2208 Ouagadougou 01, Burkina Faso; 3 Department of Clinical Sciences, Liverpool School of Tropical Medicine, Pembroke Place, Liverpool, UK

**Keywords:** Malaria, Next-generation long-lasting insecticidal nets, LLINs, PBO nets, Policy analysis, Vector control, Burkina Faso

## Abstract

**Background:**

Reductions in malaria incidence in Africa can largely be attributed to increases in malaria vector control activities; predominately the use of long-lasting insecticidal nets (LLINs). With insecticide resistance affecting an increasing number of malaria-endemic countries and threatening the effectiveness of conventional LLINs, there is an increasing urgency to implement alternative tools that control these resistant populations. The aim of this study was to identify potential challenges and opportunities for accelerating access to next-generation LLINs in Burkina Faso, a country with areas of high levels of insecticide resistance.

**Methods:**

An analytical framework was used to guide the selection of interviewees, data collection and analysis. Semi-structured interviews were carried out with key informants in April 2014 in Burkina Faso. Interviews were conducted in French and English, audio recorded, transcribed and entered into NVivo 10 for data management and analysis. Data were coded according to the framework themes and then analysed to provide a description of the key points and explain patterns in the data.

**Results:**

Interviewees reported that the policy architecture in Burkina Faso is characterised by a strong framework of actors that contribute to policymaking and strong national research capacity which indirectly contributes to national policy change via collaboration with internationally led research. Financing significantly impacts the potential adoption, availability and affordability of next-generation LLINs. This confers significant power on international donors that fund vector control. National decisions around which LLINs to procure were restricted to quantity and delivery dates; the potential to tackle insecticide resistance was not part of the decision-making process. Furthermore, at the time of the study, there was no World Health Organization (WHO) guidance on where and when next-generation LLINs might positively impact on malaria transmission, severely limiting their adoption, availability and affordability.

**Conclusions:**

This study shows that access to next-generation LLINs was severely compromised by the lack of global guidance. In a country like Burkina Faso where WHO recommendations are relatively quickly adopted, a clear WHO recommendation and adequate financing will be key to accelerate access to next-generation LLINs.

**Electronic supplementary material:**

The online version of this article (doi:10.1186/s13012-016-0469-4) contains supplementary material, which is available to authorized users.

## Background

Reductions in malaria incidence in Africa are largely attributable to improved vector control, predominately the use of long-lasting insecticidal nets (LLINs), indoor residual spraying (IRS) and, to a lesser extent, larval source management (LSM) [1, 2]. LLINs are one of the most cost-effective measures against malaria [3, 4], with World Health Organization (WHO) recommending universal coverage with LLINs (defined as universal access and use) for all people at risk of malaria [5]. Currently, pyrethroids are the only class of insecticide approved for use on LLINs, and therefore the rapid increase in mosquito resistance to pyrethroids represents a serious concern [1, 6]. The loss of LLIN effectiveness would be catastrophic, jeopardising the ability to achieve malaria control and elimination goals [7].

Insecticide resistance management strategies and alternatives to conventional LLINs need to be implemented. One option is to provide access to ‘next-generation LLINs’ treated with two or more insecticides (combination LLINs), or with an insecticide and the synergist piperonyl butoxide (PBO LLINs), designed to be more effective against pyrethroid-resistant vectors. Access to new LLINs requires the ability to acquire and use them. In this study we adopt the Frost and Reich view that access is a series of logistical, economic and political processes that affect acquisition and use [8].

Two PBO LLINs (PermaNet^©^ 3.0 and Olyset Plus^©^) are currently available on the market after receiving WHOPES interim approval as standard LLINs in 2008 and 2012, respectively [9, 10]. In 2014, the WHO Vector Control Advisory Group, whose remit is to advise WHO on new forms of vector control, recognised PermaNet^©^ 3.0 as having ‘*increased bio*-*efficacy*’ compared to pyrethroid-only LLINs in areas of insecticide resistance [11]. Recommendations for evaluating next-generation nets have recently been published [11], but at the time the study was conducted, there were no normative guidelines on when and where these should be deployed. Burkina Faso is one the few countries where a PBO LLIN was deployed as part of a national campaign in 2010 and 2013 [12].

We conducted an analysis of the context, content, processes, actors, power and role of evidence in malaria vector control policymaking in Burkina Faso and of the decision to deploy PBO LLINs in a national campaign in Burkina Faso. The study aims to identify potential challenges and opportunities for accelerating access to new vector control tools in Burkina Faso.

## Methods

### Study site

Insecticide resistance is widespread in Burkina Faso [13] and, in the southwestern region of the country, the high level of resistance is reducing the activity of insecticides on conventional LLINs [14]. Furthermore, despite two LLIN distribution rounds in 2010 and 2013, and over 70 % of children under the age of 5 years reportedly sleeping under LLINs [15, 16], the national prevalence of *Plasmodium falciparum* was 61 % in children aged 6 months to 5 years in 2014 [16]. Detailed follow-up studies in different regions of the country have found no reduction in malaria rates following the 2010 distribution programme [17, 18]. It has recently been confirmed that a third of the nets procured by Le Programme d’Appui au Développement Sanitaire (PADS, the procurement department of the Ministry of Health) for the 2010 distribution were counterfeit, non-WHO Pesticide Evaluation Scheme (WHOPES)-approved LLINs, packaged as genuine WHOPES-approved LLINs [19]. The Global Fund to Fight AIDS, Tuberculosis and Malaria (GFATM) Office of the Inspector General has highlighted significant weaknesses in the procurement process for the 2010 campaign. These weaknesses were exploited by two suppliers who provided almost 2.7 million nets that were not properly treated with insecticide and reportedly caused side effects to recipients [19]. At the time the interviews for this study were conducted, knowledge of this fraud was not in the public domain and the study team was not aware of it. Approximately 1.6 million (20 %) of the LLINs distributed in 2010 were a PBO LLIN, PermaNet^©^ 3.0 (H Pates Jamet, Vestergaard Frandsen, personal communication), but there was no accompanying monitoring and evaluation plan to compare the efficacy of the PBO LLINs with conventional LLINs.

### Analytical framework

The modified analytical framework comprised of seven themes derived from all five concepts (actors, power, context, content and process) in the Walt and Gilson policy analysis framework [20], with two additional themes, availability and affordability, from the four themes in the Frost and Reich framework [8] (Table 1). Given that the ‘policy’ under review relates to the introduction of a malaria vector control tool, the themes of availability and affordability (not contained in the Walt and Gilson framework) are important. The themes of architecture and adoption from the Frost and Reich framework are the equivalent of the actors and process themes in the Walt and Gilson framework.

**Table 1:** Framework used for sampling, interview guide and data analysis

		Definition (adapted from Walt and Gilson unless otherwise indicated)
1	Context	The systemic factors such as—political system, type of economy, employment base, national and international actions/cooperation—which may have an effect on health policy.
2	Content	The content of the policy, which reflects the output of the interplay between actors, processes and context.
3	Actors	The network of institutions and individuals that influence the adoption of a new policy
4	Power	The ability to influence, and in particular to control, resources. It can be seen in a number of dimensions including decision-making [23], agenda setting [24], thought control [25], control of financial resources and access to/level of knowledge [22].
5	Policy adoption process	The way in which policies are made, i.e. initiated, developed/negotiated/formulated/endorsed. In this study, this includes the use of evidence in the policymaking process.
6	Availability	In this study, we restrict consideration of availability to ordering (i.e. choosing and procuring a next-generation LLIN). (Frost and Reich)
7	Affordability	Involves the willingness to pay for (finance) a next-generation LLIN by global organisations as they are the primary donors of vector control. (Frost and Reich)

The modified framework was used to guide the selection of relevant policy stakeholder groups for interview, develop themes for the semi-structured interview guide, and for data analysis. For the purposes of this study, the definition of policy extends beyond a broad statement of goals [21] to include individual aspects of a policy such as the use of a specific tool [22].

### Desk review

In March 2014 and March 2015, PubMed-MEDLINE, Web of Science, Global Health, Jstor and Taylor & Francis were searched for peer-reviewed literature using the following search terms: ‘Burkina Faso’, ‘malaria’, ‘malaria control’, ‘malaria prevention’, ‘vector control’, ‘policymaking’, ‘policy analysis’, ‘decision-making’ and ‘evidence-based policy’.

In addition, using the same terms, Google, Google Scholar, the Programme National de Lutte Contre le Paludisme (PNLP) website, as well as partners’ websites, were searched for relevant reports, strategies, policies and meeting minutes. The purpose of the desk review was to identify the key actors (institutions and individuals) involved in national vector control for interview, to refine the research question and semi-structured interview guide, and to supplement findings from these.

### Study participants

The identification of the study participants was a two-step process. Using the literature and the local knowledge of two Burkinabe collaborators (Mr. Traore and Dr. N’Fale), institutions that participated in national malaria vector control policymaking were identified. An initial list of 15 institutions was drawn up and one person from each was contacted to request participation. The most senior person tasked with LLIN policy, implementation, procurement and research or funding were considered to be potential interviewees. One additional interviewee was identified during the interviewing process.

Interviewees were categorised into six groups: policymakers—staff of the Ministry of Health (MoH) working as part of the PNLP; implementers—working for non-governmental organisations (NGOs) to implement malaria control projects; multilaterals—employees of United Nations technical agencies supporting malaria control; donors—including employees of organisations that finance and procure malaria control tools; researchers—those working in academia/national institutes of research; and private sector—those in the commercial for-profit sector involved in the sale of vector control tools and insecticide products. While the sample size was guided by feasibility, interviewees were selected to encompass viewpoints from all six categories.

### Semi-structured interviews

Interviews were carried out in April 2014 in Ouagadougou, Burkina Faso. All interviews were conducted by the lead researcher and Mr Traore; 12 of the 13 interviews were conducted in French by Mr. Traore and one in English by the lead researcher. The interviewers jointly reviewed the first four interviews conducted to establish consistency in data collection. The interviews followed a semi-structured, open-ended format, which was developed in English and subsequently translated into French (Additional file 1).

The semi-structured interview guide included questions on who was involved in the policymaking process; who carried the most influence and why; how vector control policies were made (including the role of evidence); and factors that influenced the availability and affordability of PBO LLINs.

All interviewees gave signed consent for participating in the audio-recorded interviews and for the use of anonymous quotes. In reporting quotes, interviewees’ roles (e.g. policymaker) are disclosed to highlight their perspective. Ethical clearance was obtained from ethics committees at Liverpool School of Tropical Medicine and Burkina Faso (Additional file 2).

### Data analysis

Translated and accuracy-checked transcripts were entered into NVivo 10 for data management and analysis through the following four steps: (i) familiarisation (reading of transcripts); (ii) coding data according to themes in analytical framework; (iii) summarising data by interviewee and themes; and (iv) synthesis of the key points in each theme and exploration of patterns in the data.

## Results

A total of 13 people were interviewed: two researchers, four policymakers, three implementers, two donors, and two multilateral agencies. Three potential interviewees (one donor and two policymakers) did not participate; one declined and two were not available. The interviewees represented five categories as no national private sector actor was identified or interviewed. However, this perspective was later captured informally through discussions with a representative of Vestergaard Frandsen. We present findings according to the analytical framework themes, including one sub-theme that emerged during interviewing, i.e. the classification of forms of power. Within each theme, we highlight barriers and opportunities for accelerated access to next-generation LLINs in Burkina Faso. Additional quotes to support the themes are presented in Additional file 3.

### Policymaking context

In Burkina Faso, the entire population is at risk of malaria. In 2013, there were approximately 3.7 million reported confirmed malaria cases and over 6000 deaths [23]. Malaria accounts for 50 % of all outpatient consultations, 57 % of hospitalisations and 46 % of deaths [24].

At the national level, the MoH (Ministère de la Santé), through the PNLP, is responsible for all health policy and strategy development, partner coordination and resource mobilisation [25, 26]. The regional and peripheral levels focus primarily on implementation activities.

**Fig. 1:**
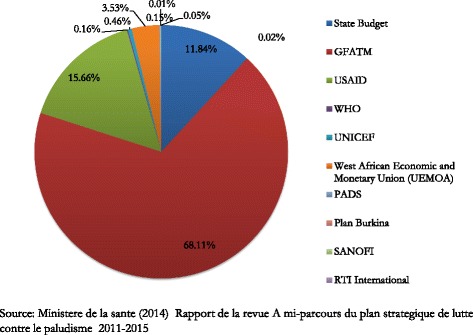
Sources of 2011 malaria control funding in Burkina Faso

As one of the world’s poorest countries [27], Burkina Faso is reliant on external organisations to finance most aspects of its malaria control interventions. In 2011, over US$ 70.6 million was spent controlling malaria [28], 68 % of which was provided by GFATM, 15 % by United States Agency for International Development (USAID), and about 12 % by the government [28]. Figure 1 shows the breakdown of 2011 expenditure on malaria control by funding source.

**Fig. 2:**
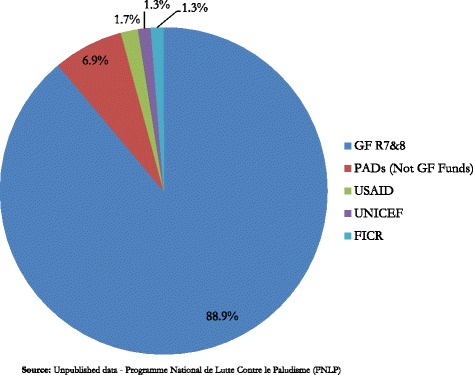
Funding source for LLINs distributed in 2010

In 2010, almost 8 million LLINs were distributed nationwide, 88.9 % were financed by GFATM through two funding rounds 7 and 8. Other sources of LLINs include those procured using donor basket funds by PADS (6.9 %) and by USAID (1.7 %), International Federation of Red Cross and Red Crescent societies (1.7 %), and UNICEF (1.3 %).^1^ Figure 2 shows the sources of support for LLINs in the 2010 LLIN nationwide distribution campaigns. In 2013, GFATM financed over 90 % of the LLINs distributed.

### National vector control policy content

The overall goal of the Burkina Faso National Malaria Strategic Plan 2011–2015 is to reduce malaria morbidity by 75 %, compared to 2010 levels, and malaria mortality to a level close to zero by the end of 2015 [28]. Table 2 summarises the vector control objectives in the Strategic Plan [28], the coverage of interventions of populations at risk as at 2012 [29] and the revised national objectives as of March 2014. In line with WHO recommendations, one objective is to achieve and maintain 100 % of the population sleeping under a LLIN by the end of 2015.

**Table 2:** Original and revised vector control policy objectives in Burkina Faso’s National Malaria Control Strategic Plan

	Vector control objectives in the 2011–2015 strategic plan [28]	Progress towards target in Burkina Faso as of 2012 [29]	Revised vector control objectives 2014 [28]	2014 Malaria Indicator Survey Results [16]
1	100 % of the population sleeping under long-lasting insecticide-treated nets (LLINs)	Approximately 50 % of total population at risk	Achieve and maintain 100 % coverage	71 % of the households have access to at least one LLIN In households with at least one LLIN, 74 % of the population of these Households slept under mosquito nets at night
2	100 % of the populations of the four health regions targeted (South-West, Cascades, Hauts-basins and Mouhoun) benefit from indoor residual spraying (IRS)	Approximately 1 % of population covered	Suspension of IRS	
3	100 % of the targeted breeding sites in the Central and Hauts-Bassins regions are covered by larviciding	No data	Extension of larviciding to Bobo-Dioulasso region	

### Policymaking actors

The interviewees identified actors involved in policymaking as the MoH and its technical departments such as PNLP; other ministries such as those for finance, communications and environment; research centres, including the Centre National de Recherche et de Formation sur le Paludisme and Centre Muraz; technical and financial partners, including WHO, GFATM, UNICEF, USAID, International Federation of Red Cross and Red Crescent societies and PLAN Burkina.

All interviewees recognised the central role of the Comité National de Pilotage de la Lutte Contre le Paludisme (Comité de Pilotage), the national steering committee for malaria, in supporting PNLP in delivering its mandate. The Comité de Pilotage is composed of five commissions, including one focused on vector control. The main responsibilities of the vector control commission are to make recommendations to PNLP in defining vector control strategies, support the development and revision of guidance documents, monitor the implementation of vector control activities, and monitor insecticide resistance [30].

**Fig. 3:**
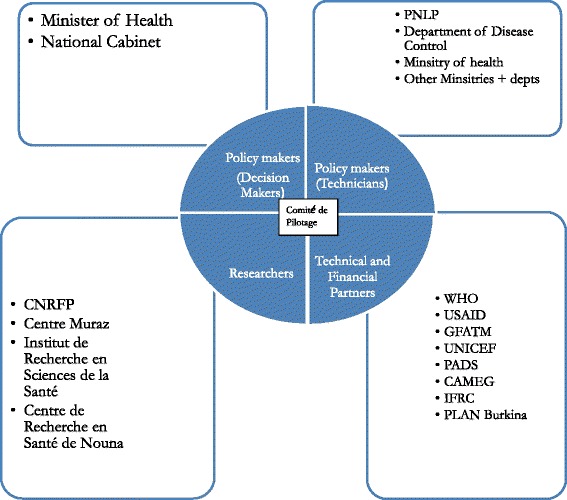
Actor and their roles in national policy making

The interviewees and the terms of reference of the Comité de Pilotage [30] outlined four main groups of actors and the roles that they played in the policymaking process (Fig. 3).

(i) Researchers: Four national research centres conduct malaria research across the country. Data generated feeds in to the national policymaking process through the MoH and Comité de Pilotage [25, 31]. However, interviewees, particularly researchers, multilaterals and donors, outlined that, while local research/researchers are valuable, there is a need to collaborate with international research institutions to strengthen the credibility of research outputs.
  ‘*Even if a research is made in Burkina Faso*, *if the signature is international it means that you have done it in collaboration with an international institute*, *this is very important. That is because in the eyes of donors*, *the international character is of great value*’ (policymaker)
(ii) Technical and financial partners: Multilaterals, donor and NGOs are collectively known as technical and financial partners. They provide technical advice and financial input to the policy development and implementation.
  ‘*The TFP* [Technical and Financial Partners] …., *they have money and they have the ideas*, *knowledge. Ok*? *Money and knowledge*’ (researcher)
(iii) Policymakers: Interviewees considered policymakers to be the technicians that drafted the policies and the decision-makers who ratified these.
(iv) Technicians: All interviewees recognised PNLP as the ‘technician’ who drafts the policy document coordinating inputs from researchers, the technical advisers and other stakeholders.
  ‘*Technical departments are really conducting daily follow*-*up*-*evaluation of the Programmes*, *which lead to new information which requires that a policy be changed. They also give a technical draft to the Office of the Ministry of Health where they decide on what needs to be done*’ (researcher)
(v) Decision-makers: All interviewees recognised the role of the national government through the MoH and the cabinet to make final decisions on ratifying policies that had been drafted by the technicians.
  ‘…*but now when the policy goes for decision making*, *the decision is taken at another level that I called politicians*; *when I say politicians. I mean the ministries*, *the Parliament who approve policies*’ (NGO)

### Perceptions of power

Power was observed when interviewees identified the actors they thought carried the most influence and when interviewees described the roles various actors played in the policymaking process.

Interviewees identified different forms of power:

(i) Power as decision-making: This dimension of power was expressed as the national government’s ability to endorse or reject a policy.
  ‘*As a technician you can write policies*, *write strategies that are relevant enough according to you*; *and they will go to the highest level for decision making*, *that*’*s something else. Those at the highest level will decide on whether they are going for this policy or not*’ (NGO)
(ii) Power to influence opinion: WHO was viewed as an important actor as its recommendations influenced national policy content and direct what donors will support, thereby mediating actor’s (e.g. PNLPs) options when drafting policy documents. National researchers also saw themselves as having a role (and to some extent influence) in contributing to the global evidence base, which in turn influences WHO recommendations.
  ‘*If the WHO recommends something*, *tomorrow you will see that people put it in application very quickly*’ (policymaker)
(iii) Financial power: All interviewees cited financial resources as being the most powerful reason for policy adoption. Consequently, GFATM who funds most of malaria control in Burkina Faso was perceived as the most influential actor [28].
  ‘*The Global Fund plays the most important role because the Global Fund is financing the malaria control programme by 80* %. *So for many policies concerning malaria control*, *the Global Fund influences much even if it is not making* [the] *decision all alone*’ (donor)

Although donors were viewed to possess a great degree of power, their power was not absolute. All interviewees recognised WHO recommendations’ influence over policy content and donor funding and two interviewees viewed decision-makers to be the most powerful actors.

### Policy adoption process

Interviewees recognised that the first step in the policy adoption of a new vector control tool would be international endorsement/recommendation of the tool.

‘*First step in* [the] *adoption process is the international adoption of the product*..… *If Burkina Faso wants to adopt a new policy*, *first of all that policy must be proved internationally*’ (NGO)

The PNLP draft the policy document with input from the partners in the Comité de Pilotage, acting in an advisory capacity. The policy is then submitted to the MoH and the national assembly for endorsement.

‘*The country adopts*; *when I say the country adopts I mean the coordinator of the national programme of malaria control must prepare the case file and submit to the hierarchy. That is to say*, *the directorate of disease control*, *the General Directorate of Health*, *the General Secretariat*, *and the Ministry. Now*, *if it is accepted*, *it becomes part of the policy*’ (policymaker)

A donor’s willingness to finance was perceived to be one of the most powerful incentives for policy adoption.

‘*Locally here what I*’*m saying is just come with your resources saying that you have money to support such strategy*, *it will be accepted*’ (researcher)

All interviewees expressed a certain level of pointlessness in going through the adoption process without global adoption and funding already being in place.

‘*The government has very few resources to put in*, *so resources are coming usually from the donors. They* [donors] *want to have it approved by the WHO first before putting their money. So meaning that you can have a very nice and promising result*, *but you need to put in place a lobby group just to push it and get it approved internationally before coming back*’ (researcher)

**Fig. 4:**
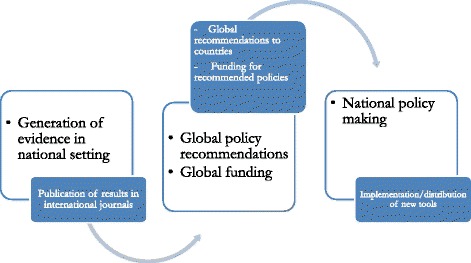
National policy adoption process for a new vector control tool

Figure 4 outlines the policy adoption process as described by interviewees showing that the genesis of the national policy process is at the global level.

### The role of evidence in vector control policymaking

There are strong formalised relationships between research centres, the MoH and Comité de Pilotage, with clear channels for communicating research results to key policymakers within the MoH and the wider stakeholders [31].

‘*At national level*, *you have the research institutions who will*, *based on the new findings*, *just report by* [MoH] *hierarchy which also transfer these findings to the Comité de Pilotage*’ (researcher)

While this should foster increased use of research in policymaking, as described above, nationally generated research was perceived to have limited impact on policy.

‘*Nationally here you have a very nice result*, *but after you finish*, *you close your reports*, *you publish*, *you go and report*, *nobody is talking about it. You have to wait to get it approved internationally and now it comes back*’ (researcher)

In this context, it is worth noting that PermaNet^©^ 3.0 was evaluated in Burkina Faso using WHOPES protocols and included in a publication describing the results of a similar trial from multiple settings [32].

#### Availability

Five brands of LLINs were distributed in 2010: Interceptor^©^, Dawa plus^©^, PermaNet^©^ (including PermaNet 2.0 and 3.0), Netprotect^©^ and Olyset^©^ net [14]. When interviewees were asked about factors influencing availability (choosing and procuring LLINs), a number of issues came to light.

Firstly, the primary factor determining LLIN availability is WHOPES recommendation.

‘*A decision maker before accepting a brand of nets must make sure that it is a net that is recognised by the WHO*; *and accepted in line with WHOPES standards*’ (NGO)

Secondly, in line with the perceptions on the importance of financial power, all interviewees noted that, for current and future vector control tools, such as next-generation LLINs, price would be a key factor in determining availability.

‘*Alright*, *money is the issue because even the Global Fund is considering the price. When you want to buy mosquito nets through the Global Fund you cannot budget for any mosquito net which costs 5 or 6 dollars each because the Global Fund could buy at 2 or 3 dollars each if they need a huge quantity of it. So the Global Fund will not accept those prices*’ (multilateral)

The factors influencing the decision to distribute a PBO LLIN (PermaNet^©^ 3.0) were explored. A few interviewees were aware that PermaNet^©^ 3.0 had been distributed but none were aware of what influenced the decision to purchase and distribute them.

‘*They provided us with that product* [PermaNet^©^ 3.0], *there*’*s no criteria on which we can ourselves choose PermaNet 3.0 just because we think resistance is lower with it than other products*’ (policymaker)

The decision to distribute PermaNet^©^ 3.0 appeared to be based on price and actions of international/external actors, i.e. net manufacturers.

‘[I was] *not aware of the decision*, [it] *looks like it was taken outside. The materials* [PermaNet^©^ 3.0] *were ordered by an external body and sent to the country*’ (researcher)

Currently, once approved by WHOPES, all brands and types of LLINs (including PBO LLINs) are considered the same. Therefore, country decisions focus on articulating specifications (physical characteristics, insecticide, binding process, dimensions etc.) to the relevant procurement department. This choice can be influenced by a desire to stick to tried and tested products or a protection of individual interests. Taken in the context of the counterfeit nets distributed in 2010, comments made by some interviewees have added significance. They seem to confirm that some actors were aware of the fraud and of the potential for high value contracts to be mismanaged for personal financial gain.

‘*So people are afraid of the unknown*, *they fear anything which is new*, *they think the other tools are already effective*, *and then they limit themselves to the old tools*, *but most often it is because some people have their own interests* [claps] *because if they import nets*, *they know what it*’*s worth. People have their own deal in the contract. It is the same people who awarded the mosquito net contracts in 2010 to their friends that we all we know*.’ (NGO)

It is worth noting that nets distributed in 2013 were procured through the GFATM voluntary pooled procurement system. Recognising weaknesses in their own practices, GFATM have also initiated a number of improvements including pre-shipment testing of nets, greater oversight of bidders’ relevant experience and the appropriateness of tender specifications, and are seeking to recover funds from those implicated in the counterfeiting [19].

#### Affordability

In Burkina Faso, the affordability of LLINs effectively means the willingness of international donors to finance it, which in turn is influenced by global recommendations.

‘*If the WHO approves the new tool*, *I believe that international initiatives will agree to finance the tool and I*’*m sure it will become more accessible*’ (policymaker)

Cost was viewed as a major influence on donors’ willingness to finance, with one interviewee citing the case of IRS being halted due to its relatively high cost.

‘T*here is not only the issue of effectiveness but also the problem of cost. Why is it that the indoor spraying which is very effective is unfortunately stopped*? *Because it is very expensive*! *It is so expensive that we cannot afford it*’ (multilateral)

A barrier to affordability, raised by all interviewees, is the need to meet set targets, which is jeopardised if the new tool is more expensive and the funding envelope remains fixed.

‘*You have set amount of money but still need to achieve universal coverage. While your target remains universal coverage because you have signed up the Abuja declaration if you want to buy more expensive nets you will have to find additional funds*.’ (multilateral)

Just one interviewee raised the issue of differences in performance between net types.

‘*Now they need to show us the methods that allow us to have more impact*, *The Global Fund is naturally interested in the impact*, *the efficiency and the effectiveness*, *and we will not get away too much from the prescribed actions at the international level in relation to a resistance that occurred*, *this for sure*.’ (donor)

Interviewees highlighted that an opportunity for improving availability and affordability would be more independence from international funding in the form of allocation of national funds to malaria control.

‘*So the first issue would be just trying to work with the policymakers to allocate resources for their own policy instead of just waiting for resources coming somewhere*’ (researcher)

## Discussion

Case studies have proven to be an effective method in exploring real-life policy events [33, 34], including examining gaps in access to drugs [35], the development of family planning programmes [36] and the coordination of donor aid policies in developing countries [37]. In Burkina Faso, much of the national policy analysis in the malaria field has focused on the adoption of artemisinin-based combination therapy and home-based management of malaria [31, 38–40]. However, Burkina Faso appears amenable to the timely translation of global guidance on malaria control into national policy; being one of the first countries to adopt intermittent preventive treatment for infants and seasonal malaria chemoprevention [29].

This is the first time the Frost and Reich framework has been used to analyse national malaria vector control policymaking in Burkina Faso. The framework and its elements, when combined with those of the Walt and Gilson framework [20], are suitable for national level policymaking analysis (recognising that sub-national factors, while important, were beyond the scope of the present study) and all responses fitted into the themes contained within the framework. However, the different dimensions of power and the different categories of policy actors are nuances within existing themes that emerged during the interviews.

A strong framework of actors linked to research centres has been identified as one of the key strengths of malaria control in Burkina Faso [28]. Burkina Faso has a strong track record of malaria vector control research with two internationally recognised research groups; they are at the forefront of insecticide resistance research with in excess of 14 publications with Burkinabe first authors on this topic in the past 10 years. More recently, a Burkina-based study was one of the first to demonstrate that standard LLIN effectiveness is compromised by insecticide resistance [14]. Despite this, the researchers interviewed felt that the ability for their outputs to influence national policy was dependent on collaboration with international researchers.

While not mentioned by any interviewees, the desk review showed that WHO’s Evidence Informed Policy Network (EVIPNet) in Burkina Faso (consisting of Burkinabe policymakers and researchers) has successfully supported evidence-based policymaking on wide-scale access to artemisinin-based combination therapies and has been pivotal in getting this funded by the GFATM [39, 41].

This study identified three dimensions of power. While only the government had power over which policies were endorsed, this was limited by its relatively low financial power. Conversely, those with financial power, such as the GFATM, are limited by the commitment to only support tools endorsed by WHO. While no actors were seen to have absolute power, financial resources conferred significant power on those that fund vector control. This is captured by the interviewees’ perception that it is futile to adopt a policy without financial backing. This finding is consistent with studies that have observed the potential for new funding to change the policymaking landscape [40] and push through policy adoption [42]. In a country like Burkina Faso, where financial power is concentrated in the hands of one institution (GFATM), the potential for scaling-up access to a new tool is tied to their willingness to finance it. This is in contrast to studies that show instances of policies being driven by actors involved in its implementation (bottom up approach) [43, 44]. This contrast is not surprising given that the LLINs are predominately delivered and financed from top down [5].

This study highlights the need for increased domestic funding for malaria control commodities [26] to reduce donor dependence [45] and increase the power of policymakers in Burkina Faso to choose appropriate interventions for their setting. Other studies have demonstrated the potential for high-level global subsidies to improve the availability of affordable high quality malaria control interventions [46]. This may be something that needs to be considered if the new vector control tools replacing those whose efficacy is being eroded by resistance have a higher unit cost.

The GFATM new funding model is a potential opportunity to improve access to new malaria control tools like next-generation LLINs. The new funding model directs up-front allocations, aligned to national strategic priorities [47]. Thus, a country like Burkina Faso would be able to make a case for the purchase of next-generation LLINs using GFATM resources, even where these are more expensive, if it were able to document reductions in the effectiveness of standard LLINs and greater effectiveness of new tools.

Vector control policymaking in Burkina Faso is largely based on policy transfer, i.e. policy ideas from one space and time influencing another [22]. The national policy process is well defined but is dependent on global malaria policymaking and available resources. Despite the recognition by the Vector Control Advisory Group of potential additional benefit of PBO LLINs against insecticide resistant mosquitoes [11], at the time the study was conducted, there was no WHO guidance on where and when next-generation LLINs might positively impact on malaria transmission, severely limiting their ability to be adopted at the national level and financed by the main donors. In December 2015, WHO Global Malaria Programme released recommendations on conditions for use of LLINs treated with PBO [48]. It recognised that PBO LLINs have increased efficacy in certain settings but argued that the evidence was too limited to justify a complete switch to PBO nets in all settings. It is evident that a switch from conventional LLINs to PBO LLINs would not be appropriate in all settings as, in areas where mosquito populations remain susceptible to pyrethroids, there is no rationale to implement a product that is likely to have a higher unit cost. However, rather than provide guidelines on when and where a switch to PBO LLINs may be justified, WHO recommends pilot exploratory implementation with robust monitoring and evaluation [48]. Nevertheless, it also states that PBO LLINs should ‘*only be used where universal coverage* […] *will not be reduced*’, which means that pilot studies are only likely to be possible where PBO nets are provided free of charge or at the same price as standard LLINs.

Donor policies only permit WHOPES-recommended LLINs [49, 50] with countries having little control over the net selection. As WHOPES do not currently distinguish between PBO and conventional LLINs, the potential to tackle resistance is not part of the decision-making process in LLIN procurement in Burkina Faso. In Burkina Faso, interviewees confirmed that the donor’s procurement department or agent oversaw the competitive bidding process where the ‘cheapest’ LLIN was bought. Vestergaard Frandsen, the manufacturers of PermaNet^©^ 3.0, have since confirmed that it was offered at a ‘competitive price’ in Burkina Faso to ensure they were used in the right context (H Pates Jamet, Vestergaard Frandsen, personal communication).

The price of a next-generation LLIN was viewed by interviewees as the single most important factor in determining its affordability (i.e. willingness to purchase). This was linked to donors’ desire to get the highest LLIN coverage for a given level of expenditure which, in turn, stems from global and national targets for universal coverage with LLINs. While not suggesting that these targets should be abandoned, it is important to review them in light of the potential for insecticide resistance to reduce LLIN performance. In the absence of a clear recommendation of when and where to target next-generation LLINs, countries may be left deploying LLINs that are less effective in areas with insecticide resistance to meet coverage targets at the expense of potentially more effective solutions.

The benefits of PBO LLINs, which can be up to US$ 2.30 more expensive than standard LLINs [12], might be clearer if donors and national programmes incorporated impact measures (overall reduction in transmission) into procurement decisions and focussed on the cost-effectiveness of alternative tools (as opposed to unit cost). In order for this to make a difference, evidence-based global recommendations on when and where next-generation LLINs are likely to provide the greatest protection, and the likely magnitude of this effect, are urgently required. The current absence of global guidance on the role and cost-effectiveness of next-generation LLINs in vector control in countries with insecticide resistance is a critical barrier to donor funding and national adoption of next-generation LLINs.

### Limitations

The findings from this study confirm that national and global levels of policymaking are interlinked. In a follow on study we look at this question is addressed from a global policymaking perspective. Although participants did not identify the private sector as key to decision-making, it may have been beneficial to have included a perspective from a manufacturer of a next-generation LLIN as opposed to directing specific questions to a representative of this sector after the interviews were complete. The problem of counterfeit nets, confirmed after the interviews had been conducted, could have limited the willingness of respondents to openly discuss LLIN procurement; however, some respondents alluded to this and hence these perspectives were captured. Finally, the researcher’s ability to be the primary data collector was limited due to language barriers and the reduced access given to researchers considered ‘outsiders’ [34]. However, this limitation provided an advantage in the data analysis process where an outsider status conferred objectivity in the interpretation of results. Mr Traore is a Burkinabe working at the Centre National de Recherche et de Formation sur le Paludisme and therefore has some level of insider status [34], allowing for additional insight into the cultural context and helping establish rapport with interviewees during data collection.

## Conclusions

This study shows that access to next-generation LLINs was severely compromised by the lack of global guidance on where and when they should be deployed. In a country like Burkina Faso, where WHO recommendations are relatively quickly adopted, a clear WHO recommendation is the key to unlocking financial resources for and accelerating access to next-generation LLINs. It remains to be seen whether the December 2015 WHO recommendation will impact on access to these products.

Furthermore, evidence collected by national research institutions on insecticide resistance should be extended to monitor (changes in) effectiveness of standard LLINs. As well as supporting evidence-based national policymaking, these data should be given greater credence in funding applications to key donors and in global policymaking.

## Abbreviations

GFATM, Global Fund to Fight AIDS, Tuberculosis and Malaria; IRM, insecticide resistance management; IRS, indoor residual spraying; LLIN, long-lasting insecticide-treated net; LSM, larval source management; MoH, Ministry of Health; NGO, non-governmental organisation; NMCP, national malaria control programme; PBO LLINs, piperonyl butoxide—long-lasting insecticide-treated net; PNLP, Programme National de Lutte Contre le Paludisme; RBM, roll back malaria; UNICEF, United Nations Children’s Fund; USAID, United States Agency for International Development; WHO, World Health Organization; WHOPES, WHO Pesticide Evaluation Scheme

## Additional files

Additional file 1: Semi-structured interview guide. (DOCX 22 kb)

Additional file 2: Ethics Approval Burkina Faso. (DOCX 1312 kb)

Additional file 3: Supplementary Quotes. (DOCX 115 kb)

## Footnotes

1 Unpublished data Programme National de Lutte Contre le Paludisme (PNLP).
