# Supplementary material for: Challenges and opportunities associated with the introduction of next-generation long-lasting insecticidal nets for malaria control: a case study from Burkina Faso
Source: Implement Sci. 2016 Jul 22;11:103. doi: 10.1186/s13012-016-0469-4 (PMC4957273; doi:10.1186/s13012-016-0469-4)
Supplement: Supplementary file 1 — Semi-structured interview guide. (DOCX 22 kb) [file 13012_2016_469_MOESM1_ESM.docx]

**Semi-Structured Interview guide**

| **Framework Themes^[[1]](#footnote-1)^** | **SSI questions in English** | **SSI questions in French** |
| --- | --- | --- |
| National architecture for coordinating access to combination LLINs (Themes: actors, power and processes) | **National architecture for coordinating access to combination LLINs** |  |
| Actors | Who are the key actors involved in coordinating access to insecticide resistance management tools like combination LLINs? (Prompt: key policymakers, financers, manufacturers) | Qui sont les principaux acteurs impliqués dans la coordination de l'accès aux outils de gestion de la résistance aux insecticides comme combinaison MILDA (: les principaux décideurs, les financiers, les fabricants) |
| Power | Which player / players would you say carries the most influence? Why? | Quel joueur / joueurs diriez-vous porte le plus d'influence? Pourquoi? |
| Process | Please describe the process of making policies in vector control | S'il vous plaît décrire le processus de rendre les politiques dans la lutte antivectorielle |
| National availability of combination LLINs (Theme Availability) | **National availability of combination LLINs** |  |
|  | Please describe the factors that led to the adoption/distribution of of permanet 3) Prompts for factors: Solution to a perceived problem, availability of funding, evidence (local/international) of efficacy), political will from superior officers. | S'il vous plaît décrire les facteurs qui ont conduit à l'adoption / distribution de PermaNet 3 Aller facteurs: Solution à un problème perçu, la disponibilité du financement, la preuve (local / international) de l'efficacité), la volonté politique des officiers supérieurs. |
|  | What would you describe as barriers to the availability of IRMT like combination LLINs? | Que feriez-vous décrire comme des obstacles à la disponibilité de la combinaison de MILDA |
|  | What/if any opportunities exist for overcoming the barriers you described? | Qu'est / si des possibilités existent pour surmonter les obstacles que vous avez décrits? |
| National affordability of combination LLINs (Themes: Affordability) | **National affordability of combination LLINs** |  |
|  | What factors in your view positively influenced the affordability of Permanet 3.0 | Quels sont les facteurs dans votre vue influence positive sur l'abordabilité des Permanet 3.0 |
|  | What would you describe as barriers to the affordability of LLINs that seek to tackle resistance (prompts: budget/finance constraints, competing demands/priorities, perceived value compared to alternative products/interventions) | Que feriez-vous décrire comme des obstacles à l'accessibilité des moustiquaires imprégnées qui cherchent à lutter contre la résistance (invites: contraintes de budget / finances, des demandes concurrentes / priorités, la valeur perçue par rapport aux produits / interventions alternatives) |
|  | What/if any opportunities existing for overcoming the barriers you described? |  |
| National adoption of Combination LLINs (Theme: Adoption) | **National adoption of combination LLINs** |  |
|  | What would you describe as barriers to the adoption of LLINs that seek to tackle resistance | Que feriez-vous décrire comme des obstacles à l'adoption de MILDA qui cherchent à attaquer la résistance |
|  | What/if any opportunities existing for overcoming the barriers you described? | Qu'est / si des opportunités existantes pour surmonter les obstacles que vous avez décrits? |
|  | Could you prioritize the top 3 issues which in your view need to be addressed in order to accelerate access to IRMT like combination LLINs at the national level | Pourriez-vous prioriser le top 3 des questions qui, à votre avis, doivent être abordés afin d'accélérer l'accès de combinaison de MILDA au niveau national |

1. Information on the 7^th^ framework theme (context) is gathered from desk review [↑](#footnote-ref-1)
