# Supplementary figures and images for: Challenges and opportunities associated with the introduction of next-generation long-lasting insecticidal nets for malaria control: a case study from Burkina Faso
Source: Implement Sci. 2016 Jul 22;11:103. doi: 10.1186/s13012-016-0469-4 (PMC4957273; doi:10.1186/s13012-016-0469-4)

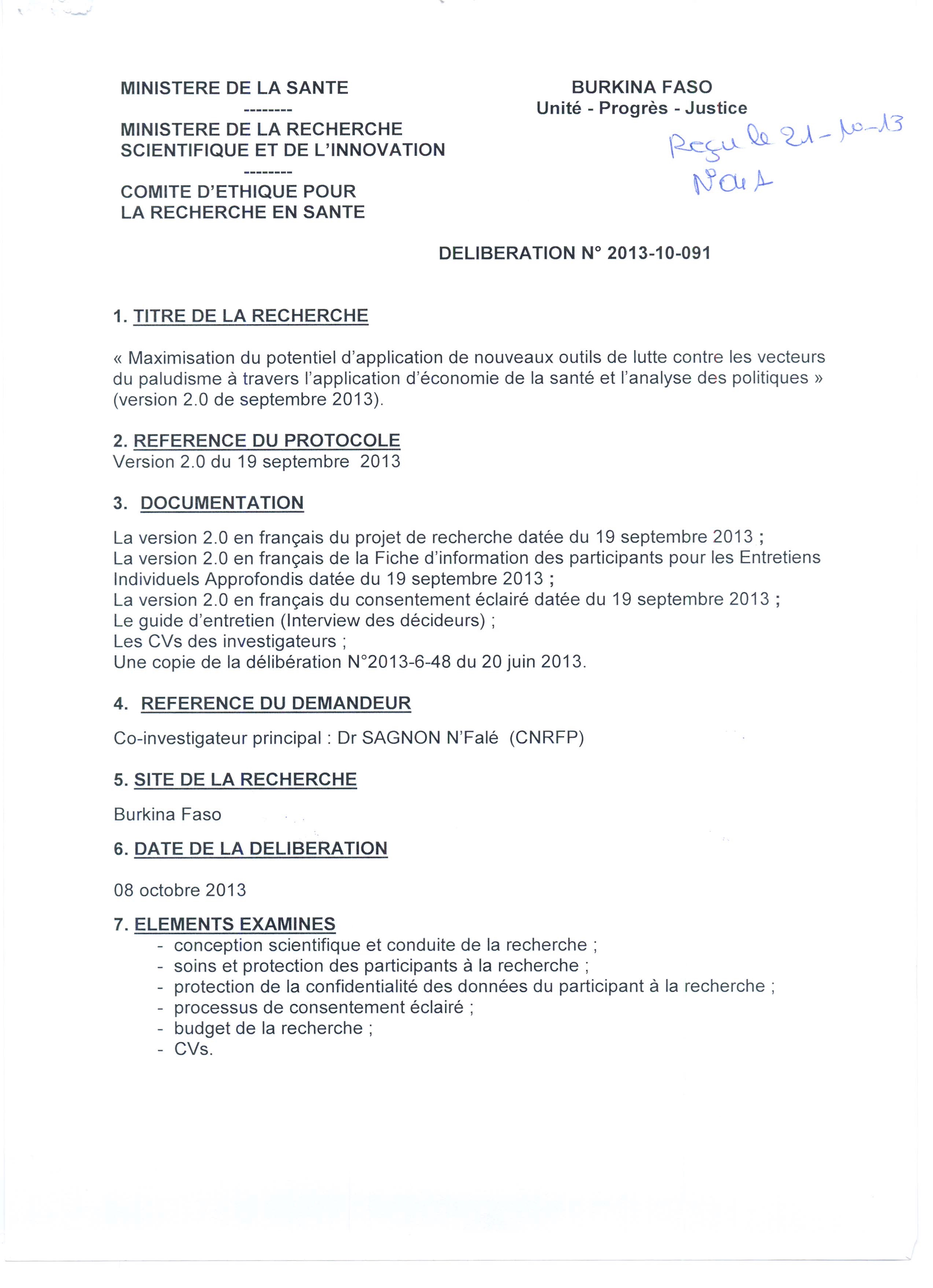

Supplement: Supplementary file 2 — Ethics Approval Burkina Faso. (DOCX 1312 kb) [file 13012_2016_469_MOESM2_ESM.docx]
