# Supplementary material for: Challenges and opportunities associated with the introduction of next-generation long-lasting insecticidal nets for malaria control: a case study from Burkina Faso
Source: Implement Sci. 2016 Jul 22;11:103. doi: 10.1186/s13012-016-0469-4 (PMC4957273; doi:10.1186/s13012-016-0469-4)
Supplement: Supplementary file 3 — Supplementary Quotes. (DOCX 115 kb) [file 13012_2016_469_MOESM3_ESM.docx]

| **SN** | **Research Finding** | **Supplementary illustrative quote** |
| --- | --- | --- |
| 1 | ***Policy adoption process***  The PNLP drafts the policy document and politicians at the highest level endorse it. | *“To summarise, … it is up to the politicians to decide, especially those who are at the ministry level, at the National Assembly (Parliament), it is up to them to decide; yes we are going for a particular strategy, no we do not go.” (NGO)* |
| 2 | ***Policy adoption process***  Policies are developed by PNLP with input from partners. | *“It is at the development stage that the technical department in charge of the issue will consult the other partners like us, the WHO, the UNICEF, all these partners are already involved in the development of the draft.”* (NGO) |
| 3 | ***Policy adoption process***  Policymakers as Technicians | *“There are technicians first who work in the shade. I mean the national program for malaria control (PNLP). These technicians draft all... strategies about malaria control.” (NGO)* |
| 4 | ***Perceptions of power***  Power as to influence opinion | *In different countries they [WHO] act as a Special Advisor to the Ministry of Health, and most of the strategies put into practice to counter lots of diseases are, to a certain extent, dictated by the WHO, who conducts countless studies and offers solutions to countries….And in general, WHO is also the organisation that provides guidance to various donors.”* (Donor) |
| 5 | ***Perceptions of power***  Financial Power | *Global Fund is providing a very significant financial support,* [compared to ]*the other partners provide* [who provide] *very little financial support.”* (Multilateral) |
| 6 | ***Perceptions of power***  Financial resources influence policy adoption | *“When it* [research] *is confirmed at the international level, now the funding bodies now adopt that strategy and now come to the country and suggest activities to the country saying ‘these are the new strategy that we have resources to support…if you are interested’. So based on that also, the Comité de Pilotage decides to go with that new strategy”* (Researcher) |
| 7 | National research indirectly influences national policymaking | *“I say that this is nice, you need to produce nice result, but you will change the situation not directly but indirectly.”* (Researcher) |
| 8 | *Availability*  The decision to procure PBO LLINs was not made nationally. | *‘I didn’t buy myself…. when they [the procurement agency] buy, they come with PermaNet 3.0’* (Policymaker) |
| 9 | *Availability*  Individual interests can affect choice of vector control tool | *“Do you think this decision maker will let you popularise your new technique that will prevent him from importing mosquito nets and therefore lose his contract percentage”* (NGO) |
|  | *Affordability*  WHO recommendations influences donor | *“Donors will fund, if you use a method that has been proven, which has been recognised by the WHO as a valid mandate’* (Donor) |
| 11 | *Affordability*  The need to meet set target is jeopardised if the new tool is more expensive and the resource envelope is fixed. | *“I think the first priority should be letting everybody have access first of all as a minimum and now start improving it.”*  (Researcher) |
| 12 | *Affordability*  National funding is a way to improve affordability and availability of new tools. | *“Poor availability of nets can be caused by the political commitment. It is also the absence of a national budget line; if we count only on the partners to acquire it.”* (NGO) |
